# Development of RP-HPLC, Stability Indicating Method for Degradation Products of Linagliptin in Presence of Metformin HCl by Applying 2 Level Factorial Design; and Identification of Impurity-VII, VIII and IX and Synthesis of Impurity-VII

**DOI:** 10.3390/scipharm85030025

**Published:** 2017-06-27

**Authors:** Sushant B. Jadhav, P. Sunil Reddy, Kalyanaraman L. Narayanan, Popatrao N. Bhosale

**Affiliations:** 1 Research and Development, Integrated Product Development, Dr. Reddy’s Laboratories Ltd, Bachupally, Hyderabad 500 090, Telangana, India; jadhavsb@drreddys.com (S.B.J.); sunilrp@drreddys.com (P.S.R.); kalyanaramanl@drreddys.com (K.L.N.); 2 Department of Chemistry, Shivaji University, Kolhapur 416 004, Maharashtra, India

**Keywords:** HPLC, design of experiments, full factorial design, development and validation, linagliptin and metformin HCl

## Abstract

The novel reverse phase-high performance liquid chromatography (RP-HPLC), stability indicating method was developed for determination of linagliptin (LGP) and its related substances in linagliptin and metformin HCl (MET HCl) tablets by implementing design of experiment to understand the critical method parameters and their relation with critical method attributes; to ensure robustness of the method. The separation of nine specified impurities was achieved with a Zorbax SB-Aq 250 × 4.6 mm, 5 µm column, using gradient elution and a detector wavelength of 225 nm, and validated in accordance with International Conference on Harmonization (ICH) guidelines and found to be accurate, precise, reproducible, robust, and specific**.** The drug was found to be degrading extensively in heat, humidity, basic, and oxidation conditions and was forming degradation products during stability studies. After slight modification in the buffer and the column, the same method was used for liquid chromatography–mass spectrometry (LC-MS) and ultra-performance liquid chromatography -time-of-flight/mass spectrometry UPLC-TOF/MS analysis, to identify *m/z* and fragmentation of maximum unspecified degradation products i.e., Impurity-VII (**7**), Impurity-VIII (**8**), and Impurity-IX (**9**) formed during stability studies. Based on the results, a degradation pathway for the drug has been proposed and synthesis of Impurity-VII (**7**) is also discussed to ensure an in-depth understanding of LGP and its related degradation products and optimum performance during the lifetime of the product.

## 1. Introduction

Linagliptin (LGP) is a long-acting xanthine-based dipeptidyl peptidase (DPP) inhibitor with high selectivity to DPP-4 compared to the related enzymes DPP-8 and DPP-9 [1,2] and has minimal risk of hypoglycemia due to its effect as a glucose-dependent insulin secretagogue [3]. LGP acts primarily by blocking incretin degradation, which inhibits the breakdown of glucagon-like peptide (GLP-1) and glucose-dependent insulinotropic peptide (GIP), stimulates insulin secretion, resulting in a reduction in plasma glucose, glucagon levels, and inhibition of gastric emptying [4,5,6]. Clinical studies with LGP demonstrated efficacy in reducing glycated hemoglobin (HbA1c) levels in type 2 diabetes patients while maintaining a placebo-like safety and tolerability profile. Linagliptin has an interesting pharmacokinetics (PK) profile, characterized by negligible renal elimination, due to that, no dose adjustment is necessary for patients with renal disease as well as in hepatic insufficiency and in elderly or obese patients [1,7].

Metformin hydrochloride (MET HCl) is an oral antihyperglycemic agent in the biguanide class and improves glucose tolerance in patients with type 2 diabetes mellitus, lowering both basal and postprandial plasma glucose. Its pharmacologic mechanisms decrease hepatic glucose production, decrease intestinal absorption of glucose, and improve insulin sensitivity by increasing peripheral glucose uptake and utilization [8]. Unlike sulfonylurea, MET HCl does not produce hypoglycemia, weight gain, and PK interactions also does not cause hyperinsulinemia [9,10,11,12,13,14,15]. With MET HCl therapy, insulin secretion remains unchanged while fasting insulin levels and day-long plasma insulin response may actually decrease [16]. Moreover, LGP and MET HCl are not prone to PK drug–drug interactions. Their co-administration improves blood glucose control more potently than either compound separately, without hypoglycemia and without increasing MET related gastrointestinal side effects. Linagliptin has a neutral effect on body weight as no weight increase is observed in mono therapy or in combination with MET HCl hence, a combination of LGP and MET HCl is very effective to control type-2 diabetes [17,18].

Design of experiment (DoE) has a statistics-based approach and has several advantages, to achieve predictive knowledge of the method with a little number of experiments and helps in optimization and development of the robust and rugged method. As a result of the DoE study, the method performance can be understood and improved due to knowledge of critical method parameters (CMPs), critical method attributes (CMAs), overlay plots of design space—and consequently normal operating range (NOR) and proven acceptable range (PAR) [19,20]—which will ensure optimal performance and reliability of the method and the data generated.

The LGP and LGP-MET HCl dosage forms monographs are not yet official in any of the pharmacopoeia. However, MET HCl alone—or in combination with some other products—is available in Indian Pharmacopoeia (IP) [21], British Pharmacopoeia (BP) [22], European Pharmacopoeia (Ph.Eur.) [23], and United States Pharmacopoeia (USP) [24].

A couple of articles on the synthesis and characterization of process related LGP impurities have been recently reported [25,26]. The manufacturing process of every manufacturer is different than each other and hence the process related impurities also different from each other therefore these articles are less helpful to pharma industries. Moreover, these articles did not discuss synthesis of degradation impurities i.e., Impurity-VII (**7**); and identification and characterization of Impurity-VII (**7**), VIII (**8**) and IX (**9**), as well as stability indicating analytical method for the nine impurities. Present work also describes plausible degradation pathways and applications of DoE for optimization of chromatographic conditions.

(Note: Due to the similar concept for development of the method and use of two level factorial design, the reflected sentences or texts in this article might be nearly similar as mentioned in reference [20]).

## 2. Materials and Methods

### 2.1. Reagents and Chemicals

The active pharmaceutical ingredient (API), placebo/matrix, tablets, and their impurities were received from “Dr. Reddy’s Laboratories Ltd”, (Integrated Product Development Organization IPDO, Hyderabad, India). Emparta grade potassium dihydrogen phosphate (KH_2_PO_4_), ortho phosphoric acid (OPA), acetic acid, ammonium acetate, HPLC-grade acetonitrile (ACN), and methanol (MeOH) were purchased from Merck (Mumbai, India). Dimethyl sulphoxide (DMSO) was purchased from Spectrochem, Mumbai, India. High-purity water obtained from Milli-Q water purification system (Bedford, MA, USA) was used for the study. Nuclear magnetic resonance (NMR) solvent DMSO-d_6_, D_2_O, and CD_3_OD were purchased from Cambridge Isotope Laboratories Inc., Tewksbury, MA, USA.

### 2.2. Instrumentation and Chromatographic Conditions

Liquid chromatography (LC) analysis was performed on a Waters high-performance liquid chromatograph (Waters Corporation, Milford, MA, USA) equipped with a photodiode array detector (PDA) at 225 nm using “Empower-2 and Empower-3” software (Waters Corporation, Milford, MA, USA). Separation was accomplished on Zorbax SB-Aq column (250 × 4.6 mm, 5 µm) with flow rate 1.0 mL min^−^^1^ using following gradient program (time in min/% mobile phase-B): 0/25, 8/25, 22/40, 28/45, 40/50, 50/50, 55/75, 60/75, 64/25, and 70/25. The following solutions were prepared: KH_2_PO_4_ buffer solution (0.02M) pH 3.0 adjusted with OPA used as mobile phase-A, and mobile phase-B, ACN:MeOH (90:10, *v/v*). The column temperature was maintained at 45 °C, and the injection volume was 40 µL. DoE and statistical analysis was performed using “Design-Expert^®^” software, version 9.0.1.0 (Stat-Ease, Inc., Minneapolis, MN, USA).

### 2.3. UPLC-TOF/MS, LC-MS and NMR Conditions

To identify *m/z* and fragmentation of maximum unspecified degradation products, High-Resolution Mass Spectroscopy (HRMS) analysis was performed on Waters Acquity TOF/MS (Waters Corporation, Manchester, UK) which was connected to UPLC via an electrospray ionization interface (ESI). Separation was accomplished on Acquity BEH shield RP18 column (100 × 2.1 mm, 1.7 µm) (Waters corporation, Milford Massachusetts, USA) with flow rate 0.21 mL min^−^^1^ and injection volume 4 µL, ammonium acetate buffer (pH 3.8 adjusted with acetic acid) as mobile phase-A, while ACN:MeOH (90:10, *v/v*) as mobile phase-B using the following gradient program (time in min/%mobile phase-B): 0/25, 1.28/25, 6.88/40, 9.28/45, 14.08/50, 18.08/50, 20.08/75, 22.08/75, 23.68/25, and 26.08/25. HPLC method is converted to UPLC with Waters Acquity column calculator software.

LC-MS analysis was performed on Synapt G2-Si LC-MS (Waters Corporation, Manchester, UK) and Zorbax SB-Aq column (250 × 4.6 mm, 5 µm) with flow rate 1.0 mL min^−^^1^ using the following gradient program (time in min/%mobile phase-B): 0/25, 8/25, 22/40, 28/45, 40/50, 50/50, 55/75, 60/75, 64/25, and 70/25. Ammonium acetate buffers (pH 3.8 adjusted with acetic acid) were used as mobile phase-A, while ACN: MeOH (90:10, v/v) as mobile phase-B with column oven temperature maintained at 45 °C.

The ESI source was operated in positive ion mode with the capillary voltage at 3.0 KV, source and desolvation temperature were set at 120 °C and 500 °C, respectively. The cone and desolvation gas flows were 60 and 800 L h^−1^, respectively.

Leucine-enkephalin was used as the lock mass generating [M+H]^+^ ion (*m/z* 556.2771) at a concentration of 200 pg mL^−1^ and flow rate 50 µL min^−1^ to ensure accuracy during analysis. The lock spray interval was set at 10 s. All data collected in centroid mode using ‘MassLynx’ software (Waters Corporation, Milford, MA, USA). The ^1^H NMR spectra were recorded on Varian Mercury plus 400 MHz FT-NMR spectrometer. The chemical shifts were reported in *δ* (ppm) relative to TMS as an internal standard.

### 2.4. Preparation of Standard, System Suitability, and Sample Solution

Linagliptin (LGP) standard stock solution was prepared by dissolving 80 mg (800 µg mL^−^^1^) in 70 mL of diluent and diluted to 250 mL with diluent (Diluent is mobile phase-A:MeOH, 50:50 *v/v*). Similarly, 0.2 µg mL^−^^1^ standard solution was prepared by appropriate dilution of stock solution with diluent.

Weighed and transferred accurately about 1.25 mg of Impurity-VII (**7**) and 1.25 mg of Impurity-IX (**9**) into a 100 mL volumetric flask, and dissolved in 5 mL of DMSO and added about 50 mL of diluent and then accurately transferred about 1.25 mg of Impurity-I (**1**) and about 1.25 mg of Impurity-II (**2**) standard into the same 100 mL volumetric flask, and sonicated it until dissolution. Diluted to volume with diluent and mixed well. Transferred 0.5 mL of above stock and 4.0 mL of standard stock solution into 25 mL volumetric flask and diluted to volume with diluent and mixed well. The solution was used to verify the system suitability by checking resolution between Impurity-VII (**7**) and Impurity-I (**1**) (**R1**); Impurity-I (**1**) and LGP (**R2**), and retention time of Impurity-II (**2**) and Impurity-VIII (**8**).

About 20 tablets of LGP-MET HCl were crushed to a fine powder with mortar and pestle. The equivalent to 5 mg of LGP was transferred to 100 mL volumetric flasks and 40 mL of diluent added after which the flasks were shaken on a rotary shaker (Lab Companion SK-600 bench top shaker, GMI, Ramsey, MN, USA) at 200 RPM for about 30 min. Then, 30 mL of diluent was added and the flask was sonicated for about 30 min with intermittent shaking. Subsequently, the solution was diluted to 100 mL with diluent and mixed well and centrifuged at 4000 RPM (about 2683× *g*) in a centrifuge machine for about 10 min (final concentration 50 µg mL^−^^1^).

## 3. Results and Discussion

### 3.1. Method Development and Optimization

We aimed to develop the RP-HPLC method to separate all process related and degradation impurities from each other and from LGP; by selecting KH_2_PO_4_ buffer, MeOH and ACN were used in the mobile phase for selectivity, while a wavelength of 225 nm was selected with maximum absorption at 227 nm.

During development stage, KH_2_PO_4_ buffer (0.02M pH adjusted to 3.0 with OPA:MeOH 90:10 *v/v*) was selected as mobile phase-A, and ACN:Water:MeOH (70:15:15 *v/v/v*) was selected as mobile phase-B with 45 °C column oven temperature. The gradient program, (time in min/%mobile phase-B): 0/25, 8/25, 30/55, 50/75, 55/75, 60/25, and 65/25 was finalized. However, during stability studies, an unknown impurity (later named as Impurity-VII (**7**)) merged with Impurity-I (**1**). Hence, to resolve the merging of Impurity-VII (**7**) and Impurity-I (**1**) issue, gradient program and mobile phase were changed to gradient program (time in min/%mobile phase-B): 0/25, 8/25, 25/40, 33/45, 38/45, 47/55, 57/80, 62/80, 65/25, and 70/25; with KH_2_PO_4_ Buffer solution (0.02M) pH 3.0 adjusted with OPA and used as a mobile phase-A, while ACN: MeOH (90:10, *v/v*) as mobile phase-B, with 45 °C column oven temperature in the above experiment, Impurity-VII (**7**) and Impurity-I (**1**) are well separated but the placebo peak interferes with Impurity-VI (**6**). Therefore, the gradient program was finally changed to gradient program (time in min/%mobile phase-B): 0/25, 8/25, 22/40, 28/45, 40/50, 50/50, 55/75, 60/75, 64/25, and 70/25; with KH_2_PO_4_ buffer solution (0.02M) pH 3.0 adjusted with OPA and used as mobile phase-A, while ACN:MeOH (90:10, *v/v*) as mobile phase-B, with 45 °C column oven temperature. It was determined that best resolution between all impurities was obtained with above finalized gradient program. To check the relation between CMA and CMP; DoE experiments were carried out using above finalized chromatographic conditions. 

### 3.2. Design of Experiments and Data Analysis

During optimization of method conditions; composition of acetonitrile in mobile phase-B (A), pH of phosphate buffer (B) and flow rate (C) were identified as CMPs. The resolution between Impurity-VII (**7**) and Impurity-I (**1**) (**R1**); and Impurity-I (**1**) and LGP (**R2**); were identified as the CMAs. Full factorial design (with 2 level and 3 variables) was selected for DoE findings using “Design-Expert” 9.0.1.0 software. A factorial model was composed of a list of coefficients multiplied by associated factor levels and this model can be expressed as Y = β0 + β2 A + β2 B + β3 C + β12AB + β13AC + … where βn is the coefficient associated with factor *n*, and the letters, A, B, C…represent the factors in the model. Combinations of factors (such as AB) represent an interaction between the individual factors.

**Figure 1:**
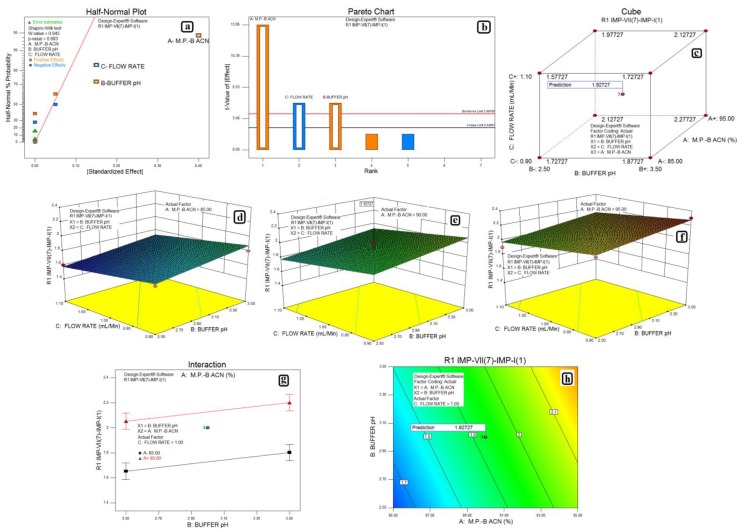
(**a**) Half-normal plot; (**b**) Pareto chart; (**c**) cube; (**d**), (**e**), and (**f**) 3D plots showing effect of % of acetonitrile (ACN) in mobile phase-B and pH of buffer on resolution R1; (**g**) effect on resolution **R1** due to interaction between buffer pH and % of ACN in mobile phase-B; and (**h**) desirability of resolution between Impurity-VII (**7**) and Impurity-I (**1**) **R1**.

**Figure 2:**
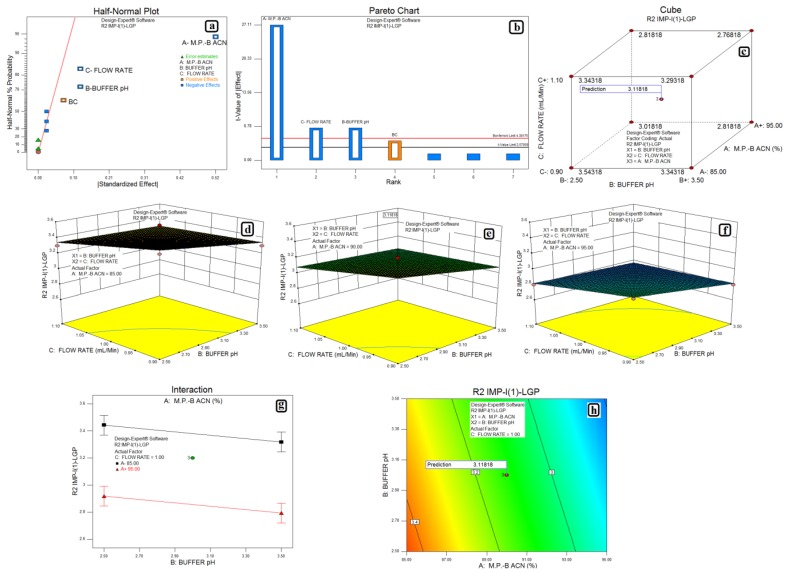
(**a**) Half-normal plot; (**b**) Pareto chart; (**c**) cube; (**d**), (**e**), and (**f**) 3D plots showing effect of % of ACN in mobile phase-B and pH of buffer on resolution R2; (**g**) effect on resolution **R2** due to interaction between buffer pH and % of ACN in mobile phase-B; and (**h**) desirability of resolution between Impurity-I (**1**) and linagliptin (LGP).

The relationship between variables was elicited using half-normal plots, Pareto charts, cube, 3D plots, interaction, and desirability plot as depicted in the Figure 1 and Figure 2; for **R1** and **R2**, respectively. In the half-normal plot, large effects (absolute values) appear in the upper-right section of the plot. Similarly, in the Pareto chart, effects above the ‘Bonferroni limit’ were almost certainly significant, while effects above the ‘*t*-value’ limit were possibly significant. Both half-normal plots and Pareto charts indicate, % of ACN in mobile phase-B (A) has more significant effect on **R1** and **R2** followed by flow rate (B) and pH of phosphate buffer (C). Positive (+ve) effect of % of ACN in mobile phase-B (A) and pH of phosphate buffer (C); while the negative effect (−ve) of flow rate (B) indicates that an increase in % of ACN in mobile phase-B (A) and pH of phosphate buffer (C) increases the resolution of **R1**; while the increase in flow rate (B) decreases the resolution of **R1**. Similarly, an increase in % of ACN in mobile phase-B (A), flow rate (B), and pH of phosphate buffer (C) decrease the resolution of **R2**. Same were confirmed with desirability and interaction plots, for **R1** and **R2**, respectively.

Further, the 3D plot shows the linear effect of % of ACN in mobile phase-B (A) (as Pareto chart) on resolution **R1** and **R2**. Desirability plot and cube predicted resolution of 1.9 for **R1** and 3.1 for **R2**, were optimum for the applied method conditions.

### 3.3. Statistical Analysis: ANOVA Analysis, Desirability and Overlay Plot

ANOVA (Analysis of variance) analysis (Table 1) showing model ‘*F*-value’ of 82.0, 208.33 for response **R1** and **R2** respectively implies the model was significant. ‘Prob > F’ values were less than 0.1 for both responses, indicates model terms were significant. The difference between adjusted and predicted R2 values were less than 0.2 as well as ‘*p*-value’ was less than 0.05 hence the model was significant. It shows a significant effect of % of ACN in mobile phase-B on resolution **R1** and **R2**.

**Figure 3:**
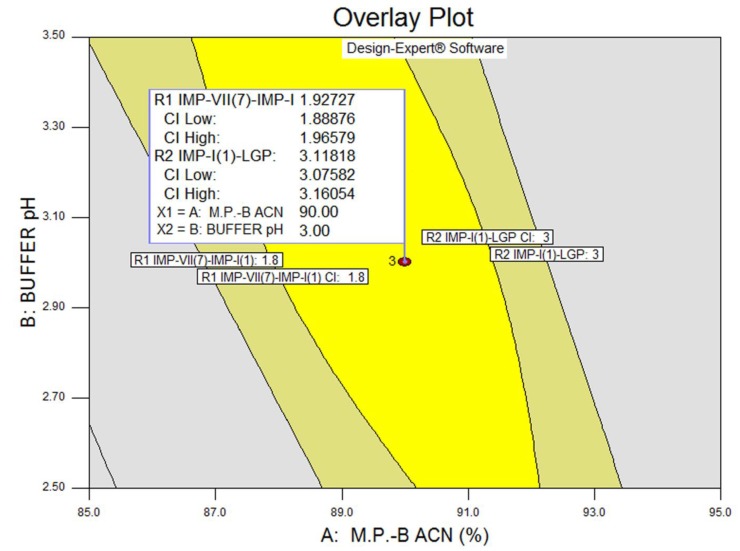
Overlay Plot with confidence interval (CI).

To achieve a resolution greater than 1.8 and 3.0 for **R1** and **R2**, % of ACN in mobile phase-B should be between 87% to 92% and the pH of the buffer should range from 2.5 to 3.4 which is shown in overlay plot (Figure 3). The PAR for the % of ACN in mobile phase-B was too low i.e., 87% to 92% which indicates that method was highly sensitive to % of ACN in mobile phase-B for **R1** and **R2**, respectively.

Therefore, the NOR for % of ACN in mobile phase-B ±2% and buffer pH ±0.2 hence it was recommended to use glass cylinders with utmost care**—**instead of plastic cylinders—while preparing mobile phase as a control strategy. The selected method parameters and chromatographic conditions gave the resolutions 1.9 and 3.1 for **R1** and **R2** respectively, which ensures minimal problems during quality control analysis. Final chromatographic conditions are as stated in 2.2 Instrumentation and Chromatographic Conditions.

### 3.4. Synthesis of Impurity-VII (**7**) and Structural Elucidation of Unspecified, Degradation Impurities

**Scheme 1:**
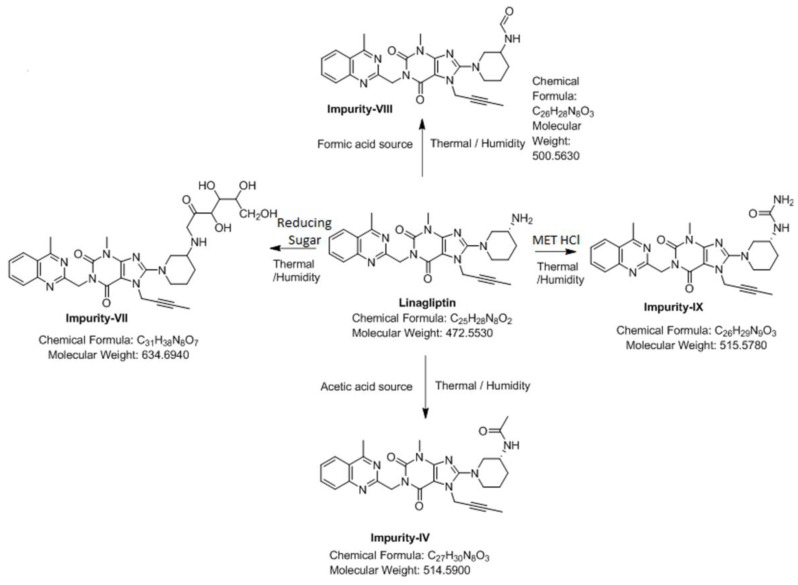
Plausible degradation pathway for linagliptin (LGP) including molecular formula and molecular weight. MET HCl: Metformin HCl.

**Scheme 2:**
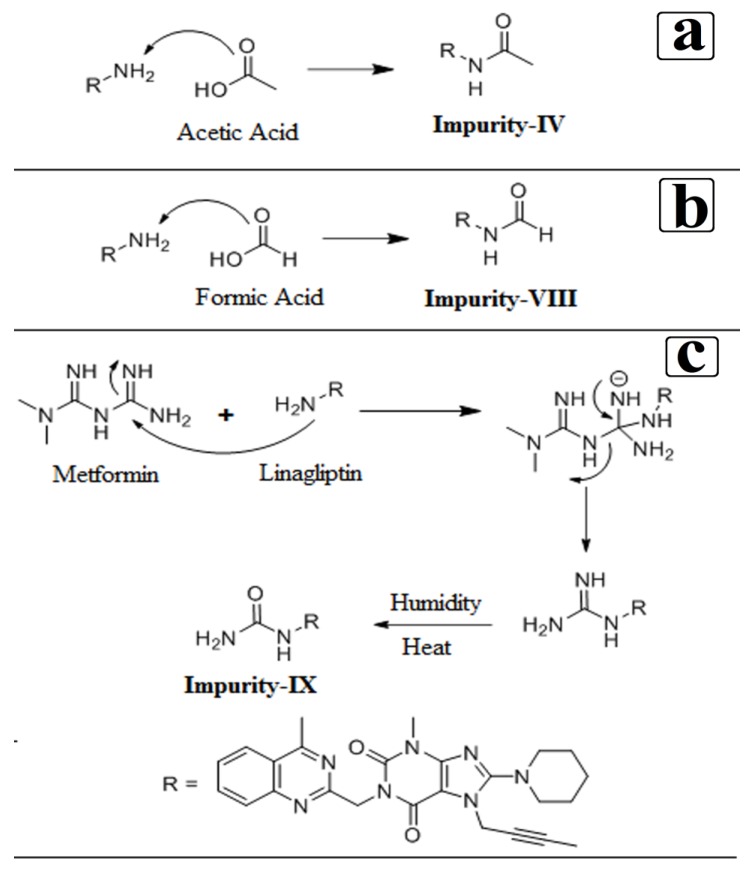
Plausible Reaction mechanism of (**a**) Impurity-IV (**4**), (**b**) Impurity-VIII (**8**), and (**c**) Impurity-IX (**9**).

**Scheme 3:**
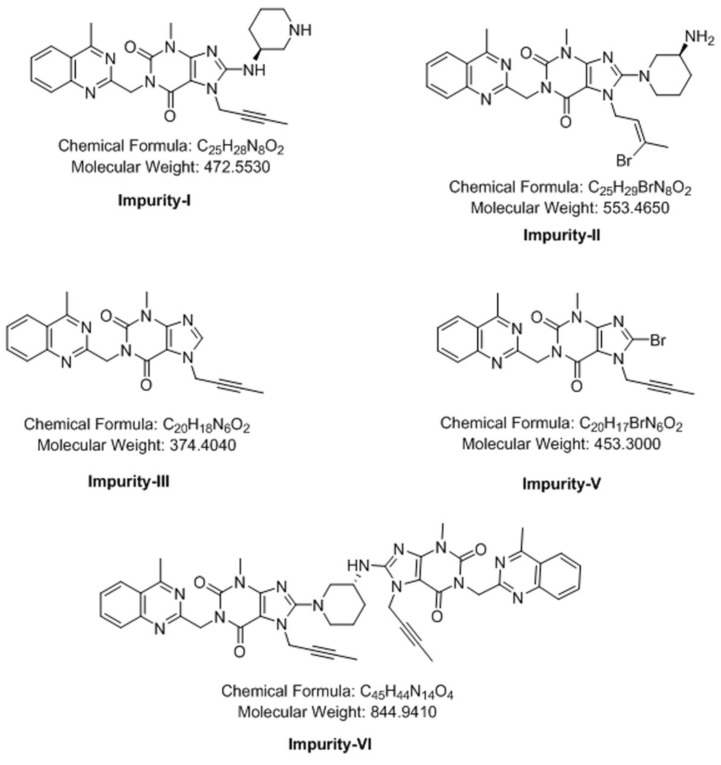
Process-related impurities including molecular formula and molecular weight.

The Impurity IV (**4**) was formed due to presence of traces of acetic acid in excipients and Impurity-VII (**7**) was formed due to presence of reducing sugar while Impurity-VIII (**8**) was formed due to the presence of formic acid in excipients and Impurity-IX (**9**) was formed in presence of MET HCl and it was observed that these four impurities will accelerate the formation in presence of heat and humidity. However, Impurity IV (**4**) and Impurity VIII (**8**) undergo a general mechanism to form amide in presence of amine and acid. The specification limits for these impurities were 1.0% and structures of these impurities were identified and confirmed by the developed method and by MS, ^1^H-NMR and Infra-Red (IR) outputs. Based on the results, degradation pathway, reaction mechanism of Impurity-IV (**7**), VIII (**8**), IX (**9**) and process related impurities were outlined as shown in Scheme 1, Scheme 2, and Scheme 3 respectively.

#### 3.4.1. Synthesis and Structural Elucidation of Impurity-VII (**7**)

Mixed 1 g of LGP and 1 g of reducing sugar in a round bottom flask and added 100 mL of toluene and refluxed for about 2 h at 120 °C, then the reaction mass was subjected to preparative HPLC for isolation of desired Impurity-VII (**7**). The reducing sugar gets oxidized and further reacts with LGP in presence of heat, humidity, and MET-HCl, forming Impurity-VII (**7**).

The IR (KBr, cm^−^^1^): 3420, 2936, 1702, 1652, 1517, 1438; ^1^H NMR (400 MHz, DMSO-d_6_): δ (ppm) 8.24–8.26 (m, 1H), 7.90–7.94 (m, 1H), 7.81–7.83 (m, 1H), 7.66–7.7 (m, 1H), 5.32 (s, 2H) 4.87 (s, 2H), 4.37–4.43 (m, 1H) 3.75–3.87 (m, 2H) 3.49–3.64 (m, 4H), 1.91–1.94 (m, 4H) 1.63–1.66 (m, 1H) 1.32 (m, 1H). MS (ESI): *m/z* 635 (M+1) and UPLC-TOF/MS spectrum showed molecular formula C_31_H_38_N_8_O_7_. The ESI-MS spectrum of the Impurity-VII (**7**) showed a molecule peak at *m/z* 635 (M+1). IR spectrum displayed the characteristic C=O absorption peak of ketone at 1702/cm. In ^1^H-NMR spectrum shows, 4 aromatic protons observed in the region of *δ* 7.7–8.24 ppm and 29 protons were observed in the region of *δ* 1.24–5.3 ppm. The reducing sugar moiety peaks were observed in the region of *δ* 1.24–5.3 ppm; based on the above spectral data the structure was identified as (*Z*)-7-(bu-2-yn-1-yl)-3-methyl-1-((4-methylquinazolin-2-yl)methyl)-8-(3-((2,3,4,5,6-pentahydroxyhex-1-en-1-yl)amino)piperidin-1-yl)-3,7-dihydro-1 H-purine-2,6-dione.

#### 3.4.2. Structural Elucidation of Impurity-VIII (**8**)

The IR (KBr, cm^−^^1^): 3453, 3363, 2939, 1699, 1660, 1566, and 1519; ^1^H NMR (400 MHz, DMSO-d_6_): *δ* (ppm) 8.24–8.26 (m, 2H), 8.02 (b,1H) 7.88–7.94 (m, 1H), 7.82–7.97 (m, 1H), 7.65–7.67 (m, 1H) 5.32 (s, 2H), 4.89 (s, 2H), 3.92–4.01 (m, 1H); 3.61–3.67 (m, 1H), 3.51–3.55 (m, 1H), 3.4 (s, 3H) 3.12–3.19 (3, 1H) 2.97–3.04 (m, 1H) 2.88 (s, 3H) 1.71–1.86 (m, 6H) 1.48–1.54 (m, 1H) MS (ESI) and UPLC-TOF/MS spectrum showed molecular formula C_26_H_28_N_8_O_3_. The ESI-MS spectrum of the Impurity-VIII (**8**) showed a molecule peak at *m/z* 501 (M+1). IR spectrum displayed the characteristic amide peak at 1699/cm. ^1^H-NMR spectrum shows, 6 aromatic protons observed in the region of δ 7.67–8.26 ppm and 22 protons were observed in the region of *δ* 1.48–5.32 ppm. The N-formylation (-NH-CHO) peaks were observed in the region of *δ* 7.67–8.26 ppm along with four aromatic protons; based on the above spectral data the structure was identified as *N*-(1-7-(but-2-yn-1-yl)-3-methyl-1-((4-methylquinazolin-2-yl)methyl)-2,6-dioxo-2,3,6,7-tetrahydro-1*H*-purin-8-yl)piperidine-3-yl)formaamide.

#### 3.4.3. Structural Elucidation of Impurity-IX (**9**)

The IR (KBr, cm^−^^1^): 3448, 2937, 1698, 1655, 1567, 1522, 1283, 1130, 946, and 762; ^1^H NMR (400 MHz, DMSO-d_6_): *δ* (ppm) 8.26 (d, 1H), 7.93–7.89 (m, 1H), 7.82–7.80 (m, 1H), 7.69–7.65 (m, 1H), 6.13 (d, 1H), 5.47 (s, 2H), 5.32 (s, 2H), 4.91 (m, 2H), 3.69–3.66 (m, 2H), 3.58–3.55 (m, 1H), 3.4 (s, 3H), 3.19 (m, 1H), 2.94–2.88 (m, 4H), 1.82–1.72 (m, 6H), 1.41–1.39 (m, 1H) MS (ESI): *m/z* 516 (M+H) and UPLC-TOF/MS spectrum showed molecular formula C_26_H_29_N_9_O_3_. The ESI-MS spectrum of the Impurity-IX (**9**) showed a molecule peak at *m/z* 516 (M+1). IR spectrum displayed the characteristic C=O absorption peak of ketone at 1698/cm. In ^1^H-NMR spectrum shows, 4 aromatic protons observed in the region of *δ* 8.26–7.65 ppm and 25 protons were observed in the region of *δ* 1.39–6.13 ppm, based on the above spectral data the structure was identified as (R)-1-(1-(7-(but-2-yn-1-yl)-3-methyl-1-((4-methylquinazolin-2-yl)methyl)-2,6-dioxo-2,3,6,7-tetrahydro-1*H*-purin-8-yl)piperidin-3-yl)urea.

### 3.5. Stress (Forced Degradation) Study/Specificity

Forced degradation and placebo interference studies were performed to demonstrate the specificity of the proposed method, by preparing placebo equivalent to the amount present in drug product as well as by preparing test and stressed with acid, base hydrolysis, peroxide oxidation, photolytic, heat, and humidity stress conditions as shown in Table 2.

**Figure 4:**
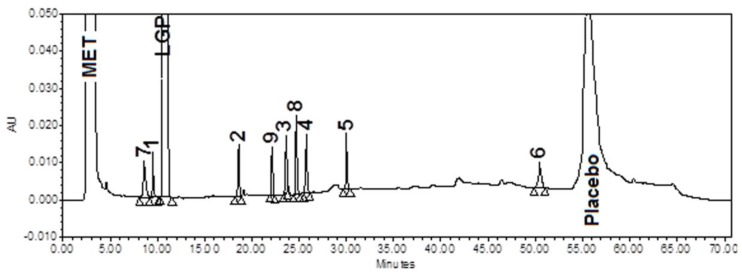
Chromatogram of impurity mix solution including identified impurities i.e., Impurity-VII (**7**), Impurity-VIII (**8**), and Impurity-IX (**9**).

Specificity study shows that all impurities are well separated from LGP as shown in Figure 4, and there was no interference at the retention time (RT) of LGP and its impurities due to diluent blank, placebo/matrix, and degradation products. Disregard the peaks due to MET HCl and its impurities up to 6 min and placebo peak at about 55 min. Mass balance for each degradation condition was between 95.8–101.3%, while the purity angle was greater than the purity threshold for each impurity and LGP in all degradation conditions, indicating the high specificity and selectivity of the method.

### 3.6. Validation

#### 3.6.1. Precision

The precision of the method was demonstrated by injecting six samples prepared by spiking test preparation with LGP impurity blend solution to get 0.5% concentration of each impurity and by spiking LGP on placebo to get 0.4% concentration. Intermediate precision was checked on a different day with different analyst, column, and system to get 0.5% concentration of each impurity. The developed method was found to be precise as the % RSD was <2.3%, for a method and <1.8%, for intermediate precision, as recommended by ICH.

#### 3.6.2. Limit of Detection and Limit of Quantitation

The signal to noise ratio method was adopted for the determination of limit of quantification. Quantification limit was achieved by injecting a series of possible dilute solutions of all impurities and LGP. The precision was also established at quantification level. The limit of detection (LOD) and limit of quantitation (LOQ) values of LGP and its impurities were less than the reporting threshold for impurities and LGP.

#### 3.6.3. Accuracy

The accuracy of the method was carried out in triplicate by adding known amount of each impurity corresponding to five concentration levels; LOQ, 50, 75, 100, and 150% of 1.0% for each impurity and LOQ, 50, 100, 125, and 150% of 0.4% of LGP along with the placebo.

The same treatment was given as described in test preparation. The percentage recoveries of all impurities at each level and each replicate were determined. The recovery of LGP and its impurities were found in the range of 95.8–112.8% with respect to added quantities of LGP and its impurities.

#### 3.6.4. Linearity and Range

The linearity was determined by preparing standard solutions at n=6 concentration levels ranging from LOQ to 300% of 0.5% for each impurity (LOQ: 0.0197, 0.0197, 0.0196, 0.0194, 0.0196, 0.0246, 0.0199, 0.0197, 0.0201, and 0.0198 μg mL^−1^ for IMP-I, II, III, IV, V, VI, VII, VIII, IX and LGP respectively). The response was linear as *r* was >0.9972 and bias at 100% response were also <0.98% for each impurity and LGP. The range was established by confirming that the analytical procedure provides an acceptable degree of linearity, accuracy, and precision when applied to samples containing amounts of analyte from LOQ to 300% of 0.5% for each impurity and 0.4% for LGP. 

#### 3.6.5. Robustness

The effect of change in flow rate (−0.1 and +0.2 mL min^−1^), column oven temperature (±5 °C), pH of mobile phase-A buffer (±0.2 pH), and mobile phase-B organic composition (±10%), on the retention time, theoretical plates, tailing factor, response ratio of first two standard injections, and % RSD were studied. It was confirmed from the system suitability results and RRTs (Relative Retention Time) that the method was robust with respect to variability in above conditions.

#### 3.6.6. Solution and Mobile Phase Stability

Stability of test and the standard solution was established on the bench top (25 °C) and in the refrigerator (2–8 °C) for 1, 2, and 5 days (d). Test solution did not show any significant change in specified, unspecified as well as on total impurities for 5 d at bench top and in refrigerator conditions. Similarity factor for standard was in the range of 0.95 to 1.05 for 5 d at bench top, as well as in refrigerator conditions, indicates that test solution and standard solution were stable for up to 5 d on the bench top and in refrigerator conditions.

Stability of mobile phase was established by storing it on the bench top (25 °C) for 5 d.

Test solution did not show any significant change with bench top stored mobile phase up to 5 d on specified, unspecified, as well as on total impurities; indicating that mobile phase was stable up to 5 days on bench top.

#### 3.6.7. Filter Compatibility

Filter compatibility was performed on Nylon and PVDF (Poly vinylidene Difluoride) 0.45 μm syringe filters. No significant change in specified, unspecified, and total impurities with respect to the centrifuged sample was observed.

From the above data, the test procedure for the related substances of LGP in LGP-MET HCl tablets was validated and found to be linear, precise, accurate, rugged, robust, specific, and stability indicating.

## 4. Conclusions

A new stability indicating reverse phase HPLC method was developed; by implementing DoE approach to understand the CMP-CMA relationship; for determination of LGP and its degradation products, for API as well as pharmaceutical dosage forms i.e., in LGP-MET HCl Tablets. The overlay plot obtained by the DoE study ensures optimum method performance over the lifetime of the product. The developed method is capable of separating nine identified and specified impurities within 70 min. Forced degradation studies were conducted, and the major degradants (i.e., Impurity-VII (**7**) Impurity-VIII (**8**), and Impurity-VIII (**9**)) formed during stability were identified using LC-MS, TOF/MS, NMR, and IR techniques; which are not yet official in any pharmacopoeia; present work also describes the synthesis of Impurity-VII (**7**), which will be helpful to pharmaceutical industry. The developed method was fully validated in accordance with ICH guidelines and found to be accurate, precise, reproducible, robust, and specific; confirming the stability indicating nature of the method. Thus, the method can be used for the “Quality Control” release of LGP API and LGP-MET HCl pharmaceutical formulations.

## Figures and Tables

**Table 1:** ANOVA analysis for selected factorial model-used for prediction and diagnostic plots (Partial sum of squares-Type-III)

Response	Source	Sum of Squares	Degrees of Freedom	Mean Square	*F* Value	*p*-Value Prob > *F*	Model Status	R^2^	Adjusted R^2^	Predicted R^2^
Impurity-VII (**7**) and Impurity-I (**1**)	Model	0.41	3	0.14	82.00	<0.0001	Significant	0.9280	0.8971	0.8300
	*A-M.P.-B:ACN*	*0.32*	*1*	*0.32*	*192.00*	*<0.0001*				
	*B-Buffer pH*	*0.045*	*1*	*0.045*	*27.00*	*0.0020*				
	*C-Flow Rate*	*0.045*	*1*	*0.045*	*27.00*	*0.0020*				
	Residual	0.010	6	0.00166						
	*Lack of Fit*	*0.010*	*4*	*0.00250*						
	*Pure Error*	*0.000*	*2*	*0.000*						
	Cor total	0.44	10							
Impurity-I (**1**) and LGP	Model	0.62	4	0.16	208.33	<0.0001	Significant	0.9522	0.9204	0.8603
	*A-M.P.-B:ACN*	*0.55*	*1*	*0.55*	*735.00*	*<0.0001*				
	*B-Buffer pH*	*0.031*	*1*	*0.031*	*41.67*	*0.0013*				
	*C-Flow Rate*	*0.031*	*1*	*0.031*	*41.67*	*0.0013*				
	Residual	0.00375	5	0.00075						
	*Lack of Fit*	*0.0037*	*3*	*0.00125*						
	*Pure Error*	*0.000*	*2*	*0.000*						
	Cor total	0.66	10							

Cor Total: Totals Corrected for the mean; M.P.-BACN: Mobile Phase-B:Acetonitrile; LGP: Linagliptin.

**Table 2:** Purity Angle, Purity Threshold, %Degradation, Mass Balance, and Purity Flag obtained for each stress conditions

Nature of Stress	Stress Condition	Purity Angle	Purity Threshold	%Degradation	Mass Balance	Purity Flag
Acid	10 mL-5N HCl- for 23 h on bench top	0.146	0.267	0.1117	99.7	No
Base	10 mL-5N NaOH- for 25 h on bench top	0.192	0.320	3.3189	96.2	No
Peroxide	20 mL 30% H_2_O_2_-2 hon bench top	0.137	0.319	1.6259	95.8	No
Water	10 mL of water 5 days on bench top	0.144	0.262	Nil	99.7	No
Thermal	At 105 °C for 6 h in an oven	0.145	0.267	1.8186	96.3	No
Photostabilty	200 watt hours/square meter and 1.2 million lux hours	0.143	0.271	0.0528	101.3	No
Humidity	90%RH at 25 °C for 7 days	0.140	0.275	0.1550	100.5	No

RH: Relative Humidity.

## Supplementary Materials

The following are available online at www.mdpi.com/2218-0532/85/3/25/s1, Figure S1: Overlaid Chromatogram of impurity mix solution, placebo, diluent blank and standard solution; Figure S2: Chromatograms showing degradation studies in unstressed, acid, base and peroxide; Figure S3: Chromatograms showing degradation studies in water, thermal, photolytic and humidity; Table S1: Design of experiment (DoE) design and results obtained by full factorial design with Design Expert Software; Table S2: Results of Precision (*n* = 6), Intermediate Precision (*n* = 6); LOD and LOQ (*n* = 6); Accuracy at LOQ (*n* = 3); Accuracy at 150% (*n* = 6); Correlation coefficient (*r*) and %Bias at 100% and Table S3: Results of forced degradation with respect to individual impurity; Tables S4, S5 and S6: NMR assignments for structural elucidation of Impurity-VII, -VIII and -IX respectively.
