# Supplementary material for: Development of RP-HPLC, Stability Indicating Method for Degradation Products of Linagliptin in Presence of Metformin HCl by Applying 2 Level Factorial Design; and Identification of Impurity-VII, VIII and IX and Synthesis of Impurity-VII
Source: Sci Pharm. 2017 Jun 27;85(3):25. doi: 10.3390/scipharm85030025 (PMC5620513; doi:10.3390/scipharm85030025)
Supplement: Supplementary file 1 [file scipharm-85-00025-s001.pdf]

List of Figures (Supplementary)

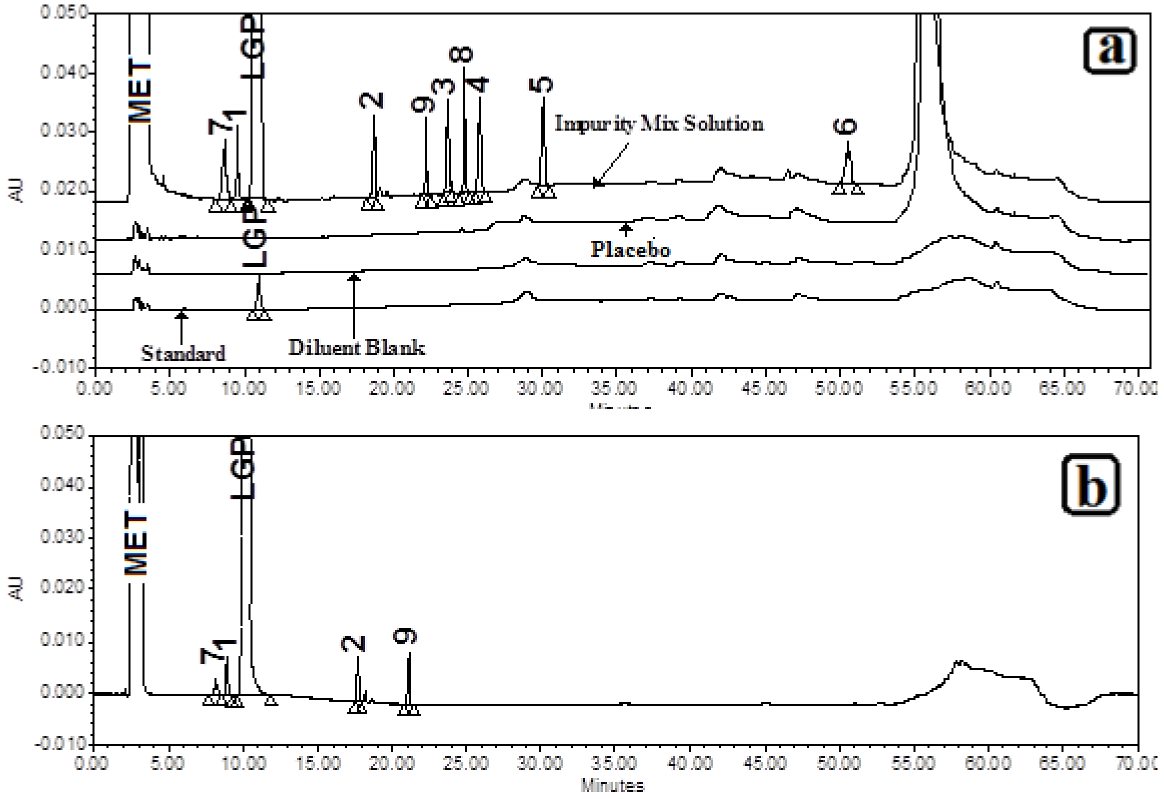

Figure S1. (**a**) Overlaid Chromatogram of impurity mix solution Impurity-I (**1**), Impurity-II (**2**), Impurity-III (**3**), Impurity-IV (**4**), Impurity-V (**5**), Impurity-VI (**6**), Impurity-VII (**7**), Impurity-VIII (**8**), Impurity-IX (**9**), placebo, diluent blank and standard solution (**b**) System suitability solution.

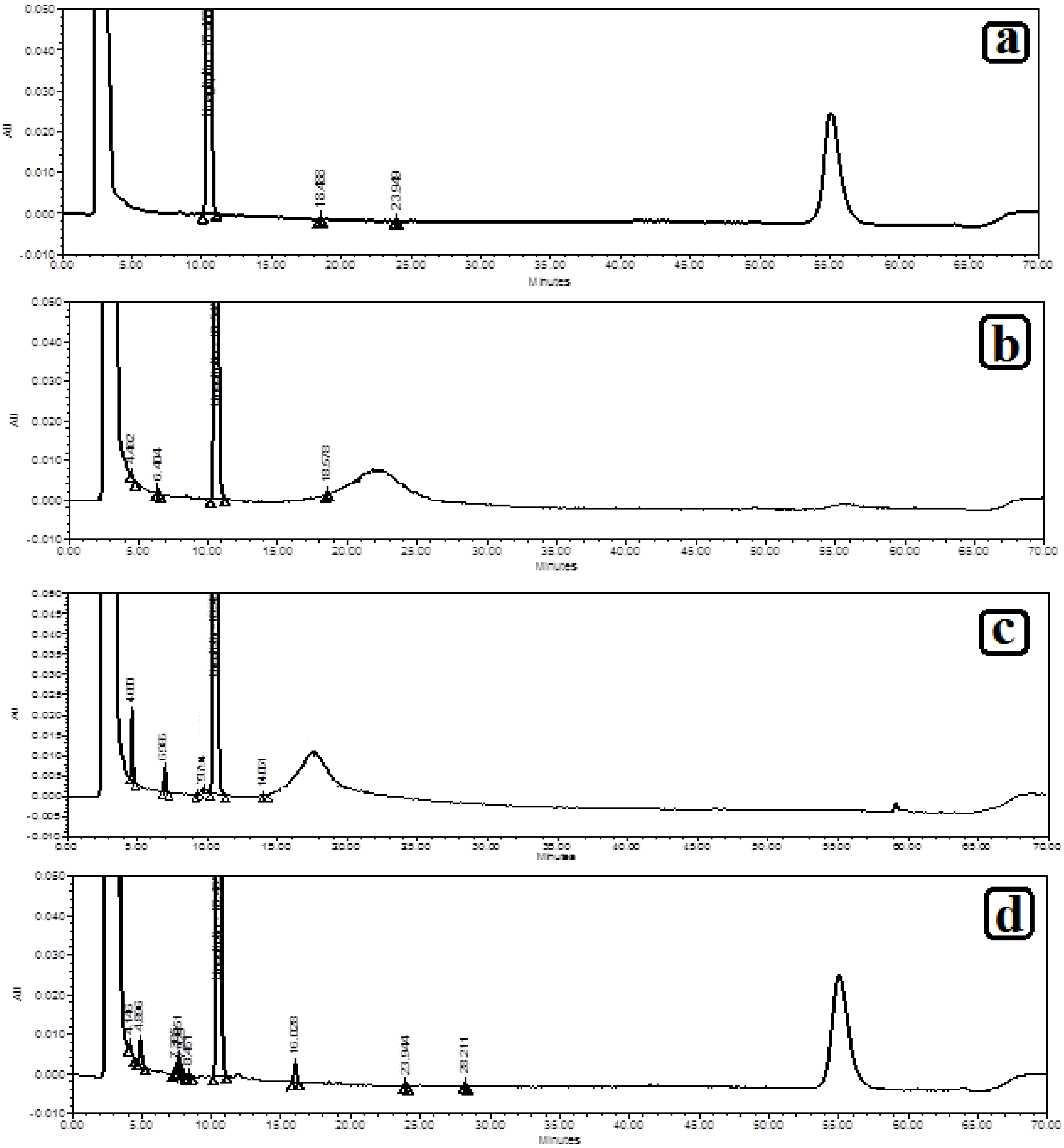

Figure S2. Chromatograms showing degradation studies in (**a**) Unstressed; (**b**) Acid; (**c**) Base and (**d**) Peroxide.

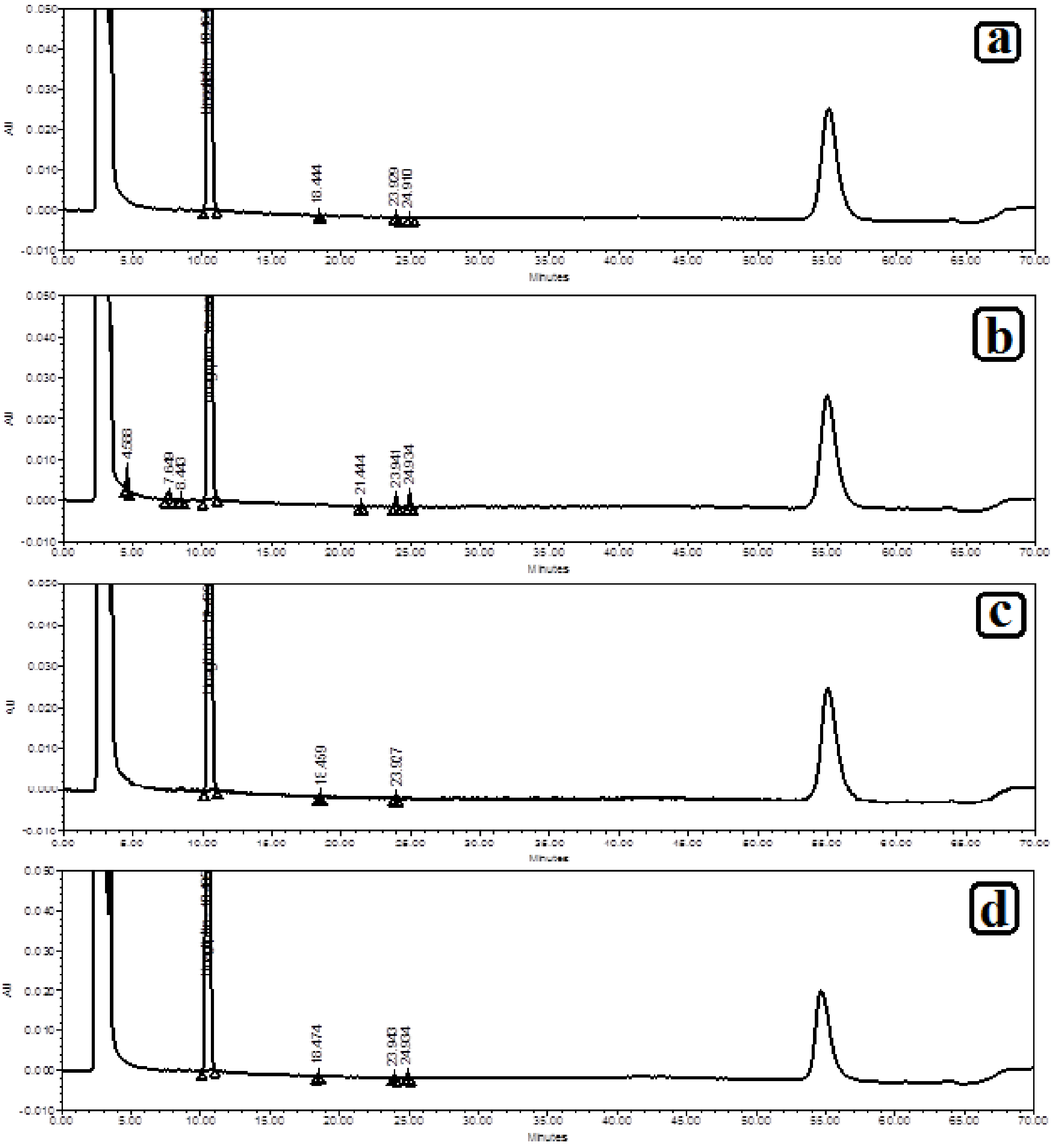

Figure S3. Chromatograms showing degradation studies in (**a)** Water; (**b**) Thermal; (**c**) Photolytic and (**d**) Humidity.

Tables (Supplementary)

Table S1. Design of Experiment (DoE) design and results obtained by full factorial design with Design Expert Software

Std	Run	Type	Factor 1	Factor 2	Factor 3	Resolution -1 (R1)	Resolution-2 (R2)
			A:M.P.-B (ACN) %	B:Buffer pH	C:Flow Rate mL/Min	IMP-VII (7) and IMP-I (1)	IMP-I (1) and LGP
6	1	Factorial	95.0	2.50	1.10	1.9	2.8
4	2	Factorial	95.0	3.50	0.90	2.3	2.8
9	3	Center	90.0	3.00	1.00	2.0	3.2
8	4	Factorial	95.0	3.50	1.10	2.1	2.7
3	5	Factorial	85.0	3.50	0.90	1.8	3.3
11	6	Center	90.0	3.00	1.00	2.0	3.2
7	7	Factorial	85.0	3.50	1.10	1.7	3.3
10	8	Center	90.0	3.00	1.00	2.0	3.2
5	9	Factorial	85.0	2.50	1.10	1.6	3.3
2	10	Factorial	95.0	2.50	0.90	2.1	3.0
1	11	Factorial	85.0	2.50	0.90	1.7	3.5

Table S2. Results of Precision (*n*=6), Intermediate Precision (*n*=6), LOD and LOQ (n=6), Accuracy at LOQ (*n*=3), Accuracy at 150% (*n*=6), correlation coefficient (*r*) and %Bias at 100%

**Parameters**	**% of LGP Impurities**									
	**(I)**	**(II)**	**(III)**	**(IV)**	**(V)**	**(VI)**	**(VII)**	**(VIII)**	**(IX)**	**LGP**
Precision (% RSD)	1.5	1.4	1.6	1.6	0.8	2.1	1.7	0.8	0.1	2.3
Intermediate Precision (% RSD)	0.5	0.8	0.2	0.2	0.6	1.1	0.4	0.3	0.1	0.6
LOD (%)	0.012	0.012	0.014	0.014	0.014	0.014	0.010	0.014	0.010	0.013
LOQ (%)	0.040	0.037	0.041	0.038	0.041	0.047	0.036	0.043	0.039	0.037
Accuracy at LOQ (%)	93.9	95.6	104.6	99.2	108.4	92.8	86.5	108.3	96.4	100.5
Accuracy at 150%	99.3	100.2	97.8	100.8	96.0	112.8	99.5	101.2	108.1	95.9
Correlation Coefficient (*r*)	0.9999	0.9999	0.9999	0.9999	0.9999	0.9999	0.9999	0.9999	0.9999	0.9972
% Bias at 100%	0.03	0.19	-0.59	0.16	0.98	0.98	0.10	0.36	0.07	0.69

Table S3 Results of forced degradation with respect to individual impurity

**Nature of Stress**	**IMP-I**	**IMP-II**	**IMP-III**	**IMP-IV**	**IMP-V**	**IMP-VI**	**IMP-VII**	**IMP-VIII**	**IMP-IX**
Unstressed	0.0273	Not Detected	Not Detected	0.0273	Not Detected	Not Detected	0.1127	0.1099	Not Detected
Acid	0.0392	Not Detected	Not Detected	Not Detected	Not Detected	Not Detected	0.1218	0.1172	Not Detected
Base	0.0701	Not Detected	Not Detected	0.0568	Not Detected	Not Detected	Not Detected	0.0827	Not Detected
Peroxide	Not Detected	Not Detected	Not Detected	0.0435	Not Detected	Not Detected	0.0495	0.1118	Not Detected
Water	Not Detected	Not Detected	Not Detected	0.0446	Not Detected	Not Detected	0.0609	0.1086	Not Detected
Thermal	0.0295	Not Detected	Not Detected	0.5331	Not Detected	Not Detected	0.1254	0.4378	Not Detected
Photolytic	0.0483	Not Detected	Not Detected	0.0372	Not Detected	Not Detected	0.1171	0.1279	Not Detected
Humidity	Not Detected	Not Detected	Not Detected	0.2238	Not Detected	Not Detected	0.0757	0.1338	Not Detected

NMR Assignments for structural elucidation of Impurity-VII (7), Impurity-VIII (8) and Impurity-IX (9)

a) NMR Assignments for structural elucidation of Impurity-VII (7)

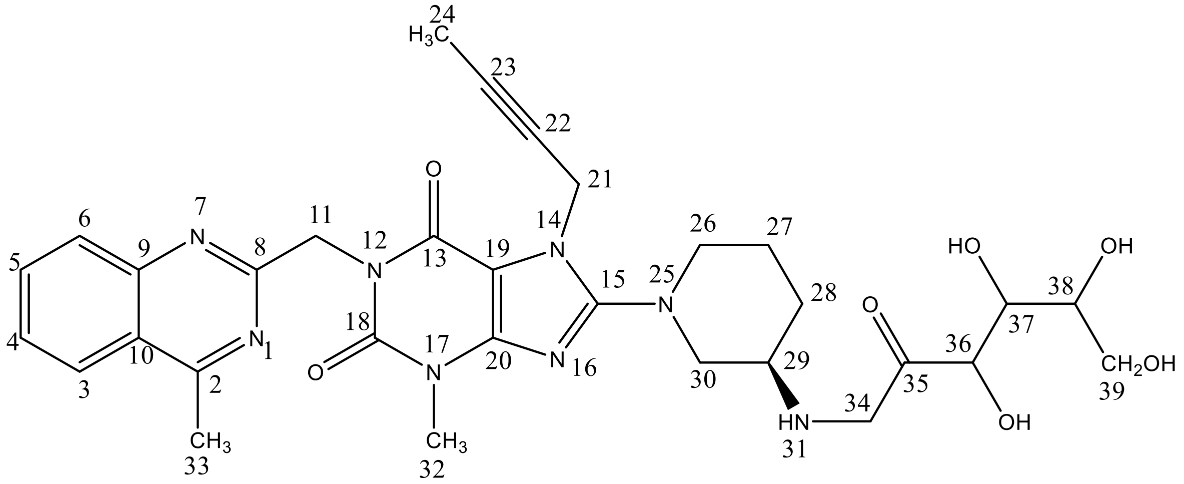

In proton NMR spectrum of impurity it was observed that one amine proton is absent. The proton count is having 11 extra protons. Unaccounted signals were observed in proton and carbon NMR spectrum indicates impure nature of compound.

Table S4 NMR assignments for Impurity-VII (7)

Position[1]	^{1}H	δ (ppm)	*J(Hz)[2]*	gCOSY	^{13}C	DEPT	gHSQC
2	-	-	-	-	168.9	-	-
3	1H	7.80	d, 8.0	(4H, 7.60)	125.7	CH	(3H, 7.80)
4	1H	7.90	t, 7.6	(3H, 8.15)<br>(5H, 7.85)	127.2	CH	(4H, 7.90)
5	1H	7.66	m	(4H, 7.60)<br>(6H, 7.80)	134.1	CH	(5H, 7.66)
6	1H	8.22	d, 8.4	(5H, 7.85)	127.9	CH	(6H, 8.22)

8	-	-	-	-	161.0	-	-
9	-	-	-	-	150.9	-	-
10	-	-	-	-	122.5	-	-
11	2H	5.32	s	-	45.5	CH_2	(11H, 5.32)
13	-	-	-	-	156.0	-	-
15	-	-	-	-	156.1	-	-
18	-	-	-	-	153.2	-	-
19	-	-	-	-	103.3	-	-
20	-	-	-	-	150.0	-	-
21	2H	4.86	m	(24H, 1.78)	35.5	CH_2	(21H, 4.86)
22	-	-	-	-	63.1	-	-
23	-	-	-	-	97.3	-	-
24	3H	1.76	s	(21H, 4.90)	3.1	CH_3	(24H, 1.76)
26	1Ha	3.16	m	(26He, 3.67)<br>(27Ha, 1.78)<br>(27He, 1.89)	50.2	CH_2	(26Ha, 3.16)
	1He	3.54	m	(26Ha, 3.10)<br>(27Ha, 1.78)<br>(27He, 1.89)	-	-	(26He, 3.54)
27	1Ha	1.70	m	(26Ha, 3.10)<br>(26He, 3.67)<br>(27He, 1.89)<br>(28Ha, 1.42)<br>(28He, 2.02)	23.2	CH_2	(27Ha, 1.70)
	1He	1.84	m	(26Ha, 3.10)<br>(26He, 3.67)<br>(27Ha, 1.78)<br>(28Ha, 1.42)<br>(28He, 2.02)	-	-	(27He, 1.84)

28	1Ha	1.54	m	(27Ha, 1.78)<br>(27He, 1.89)<br>(28He, 2.02)<br>(29H, 3.02)	33.5	CH_2	(28Ha, 1.54)
	1He	1.87	m	(27Ha, 1.78)<br>(27He, 1.89)<br>(28Ha, 1.42)<br>(29H, 3.02)	-	-	(28He, 1.87)
29	1H	2.60	m	(28Ha, 1.42)<br>(28He, 2.02)<br>(30Ha, 2.88)<br>(30He, 3.75)	54.1	CH	(29H, 3.97)
30	1Ha	2.70	m	(29H, 3.02)<br>(30He, 3.75)	55.2	CH_2	(30Ha, 2.7)
	1He	3.80	m	(29H, 3.02)<br>(30Ha, 2.88)	-	-	(30He, 3.80)
31	NH	8.22	br	-	-	-	-
32	3H	3.40	s	-	29.9	CH_3	(32H, 3.40)
33	3H	2.88	s	-	22.1	CH_3	(33H, 2.88)
34	2H	-	s		52.0	CH_2	(34H, 8.03)
35	-	-	-	-		-	-
36	1H	3.7	m	-	77.3	CH	(36H, 3.7)
37	1H	3.6	m	-	69.9	CH	(34H, 3.6)
38	1H	3.6	m	-	69.3	CH	(38H, 3.6)
39	2H	3.5	m	-	63.6	CH_2	(39H, 3.5)

[1] Refer the structural formula in previous page for numbering

[2]This column gives the ^{1}H-^{1}H multiplicity and coupling constants

s-singlet, d-doublet, t-triplet, m-multiplet.

b) NMR Assignments for structural elucidation of Impurity-VIII (8)

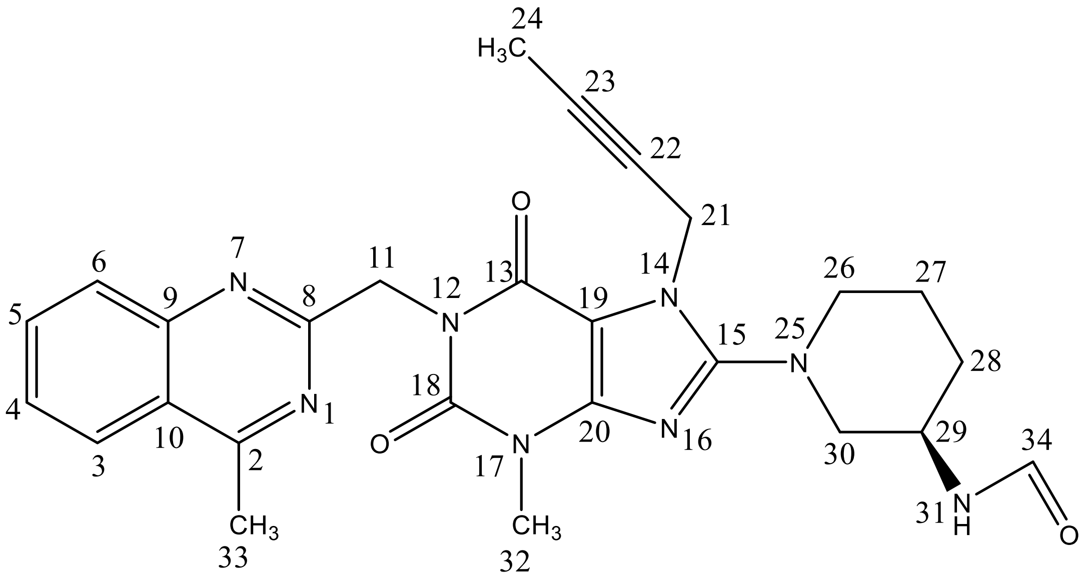

In proton NMR spectrum of impurity it was observed that one amine proton is absent. One proton at 8.0ppm was observed corresponding to proton of formaldehyde (34H). In ^{13}C NMR spectrum of impurity it was observed that one carbon signal at 160.9ppm was observed corresponding to carbon of formaldehyde (34C). In DEPT spectrum one methyl signal at 160.9ppm was observed, this indicates presence of aldehydic CH. The Heteronuclear Single Quantum Coherence (HSQC) spectrum of impurity showed correlation between 34C at 160.9ppm and 34H at 8.03ppm, indicating N-formylation at 31N position. The correlation for 29C with 31H at 8.22 ppm and 34H at 8.03ppm, through bond observed in Hetronuclear Multiple Bond Correlation (HMBC) spectrum of impurity. This indicates the bond formation between 31N and 34C (N-formaldehyde bond formation)

Table S5 NMR assignments for Impurity-VIII (8)

Position[1]	^{1}H	δ (ppm)	*J(Hz)*[2]	gCOSY	^{13}C	DEPT	gHSQC
2	-	-	-	-	171.0	-	-
3	1H	7.80	d, 8.0	(4H, 7.60)	126.7	CH	(3H, 7.80)

4	1H	7.90	t, 7.6	(3H, 8.15)<br>(5H, 7.85)	128.6	CH	(4H, 7.90)
5	1H	7.66	m	(4H, 7.60)<br>(6H, 7.80)	135.5	CH	(5H, 7.66)
6	1H	8.22	d, 8.4	(5H, 7.85)	128.9	CH	(6H, 8.22)
8	-	-	-	-	162.4	-	-
9	-	-	-	-	150.8	-	-
10	-	-	-	-	124.2	-	-
11	2H	5.32	s	-	47.2	CH_2	(11H, 5.32)
13	-	-	-	-	155.6	-	-
15	-	-	-	-	158.3	-	-
18	-	-	-	-	153.4	-	-
19	-	-	-	-	105.7	-	-
20	-	-	-	-	150.0	-	-
21	2H	4.90	m	(24H, 1.78)	36.9	CH_2	(21H, 4.90)
22	-	-	-	-	74.4	-	-
23	-	-	-	-	82.5	-	-
24	3H	1.76	s	(21H, 4.90)	3.3	CH_3	(24H, 1.76)
26	1Ha	3.16	m	(26He, 3.67)<br>(27Ha, 1.78)<br>(27He, 1.89)	51.7	CH_2	(26Ha, 3.16)
	1He	3.54	m	(26Ha, 3.10)<br>(27Ha, 1.78)<br>(27He, 1.89)	-	-	(26He, 3.54)
27	1Ha	1.70	m	(26Ha, 3.10)<br>(26He, 3.67)<br>(27He, 1.89)<br>(28Ha, 1.42)	24.8	CH_2	(27Ha, 1.70)

				(28He, 2.02)			
	1He	1.84	m	(26Ha, 3.10)<br>(26He, 3.67)<br>(27Ha, 1.78)<br>(28Ha, 1.42)<br>(28He, 2.02)	-	-	(27He, 1.84)
28	1Ha	1.54	m	(27Ha, 1.78)<br>(27He, 1.89)<br>(28He, 2.02)<br>(29H, 3.02)	33.9	CH_2	(28Ha, 1.54)
	1He	1.87	m	(27Ha, 1.78)<br>(27He, 1.89)<br>(28Ha, 1.42)<br>(29H, 3.02)	-	-	(28He, 1.87)
29	1H	3.97	m	(28Ha, 1.42)<br>(28He, 2.02)<br>(30Ha, 2.88)<br>(30He, 3.75)	48.5	CH	(29H, 3.97)
30	1Ha	3.00	m	(29H, 3.02)<br>(30He, 3.75)	58.2	CH_2	(30Ha, 3.00)
	1He	3.65	m	(29H, 3.02)<br>(30Ha, 2.88)	-	-	(30He, 3.65)
31	NH	8.22	br	-	-	-	-
32	3H	3.40	s	-	30.3	CH_3	(32H, 3.40)
33	3H	2.88	s	-	21.8	CH_3	(33H, 2.88)
34	1H	8.03	s			CH	(34H, 8.03)

[1] Refer the structural formula in previous page for numbering

[2]This column gives the ^1H-^1H multiplicity and coupling constants

s-singlet, d-doublet, t-triplet, m-multiplet.

c) NMR Assignments for structural elucidation of Impurity-IX (9)

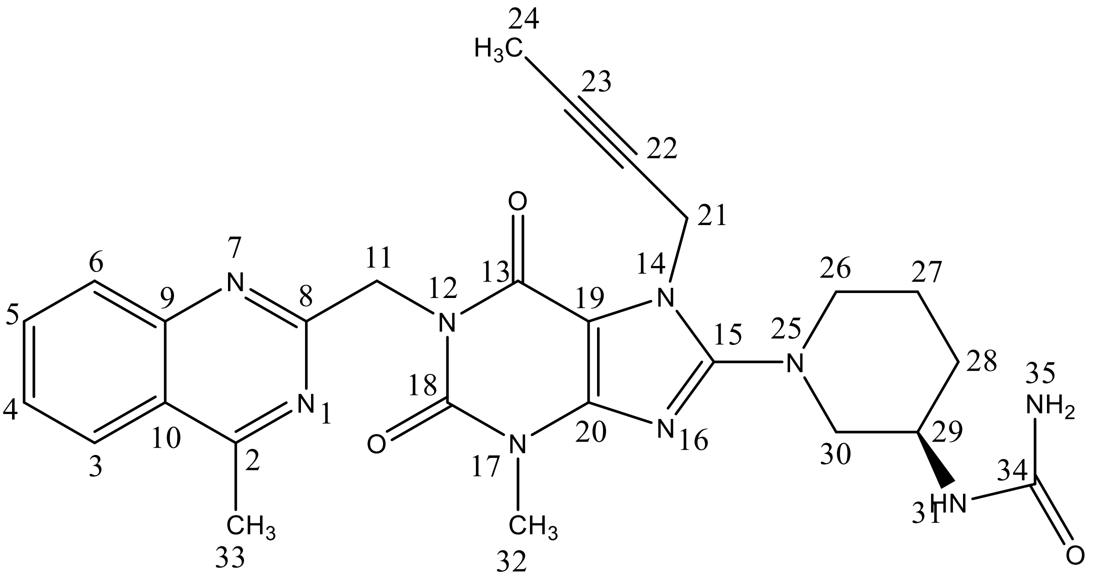

In proton NMR spectrum of impurity it was observed that one amine proton is absent. Two exchangeable protons at 5.32ppm were observed (35H). In ^{13}C NMR spectrum of impurity it was observed that one carbon signal at 161.0ppm was observed corresponding to carbon position 34C. In Hetronuclear Multiple Bond Correlation (HMBC) spectrum of impurity through bond correlation was observed for 34C at 161.0 and 35H at 5.32ppm.

Table S6 NMR assignments for Impurity-IX (9)

Position[1]	^{1}H	δ (ppm)	*J(Hz)*[2]	gCOSY	^{13}C	DEPT	gHSQC
2	-	-	-	-	168.8	-	-
3	1H	8.24	d, 7.6	(4H, 7.67)	125.8	CH	(3H, 8.24)
4	1H	7.67	t, 7.2	(3H, 8.24)<br>(5H, 7.91)	127.2	CH	(4H, 7.67)
5	1H	7.91	m	(4H, 7.67)<br>(6H, 7.81)	134.1	CH	(5H, 7.91)
6	1H	7.81	d, 8.4	(5H, 7.91)	127.9	CH	(6H, 7.81)
8	-	-	-	-	161.0	-	-

9	-	-	-	-	150.9	-	-
10	-	-	-	-	122.5	-	-
11	2H	5.32	s	-	45.3	CH_2	(11H, 5.46)
13	-	-	-	-	155.9	-	-
15	-	-	-	-	158.0	-	-
18	-	-	-	-	153.2	-	-
19	-	-	-	-	103.3	-	-
20	-	-	-	-	149.0	-	-
21	2H	4.91	m	(24H, 1.78)	35.4	CH_2	(21H, 4.91)
22	-	-	-	-	73.7	-	-
23	-	-	-	-	81.3	-	-
24	3H	1.78	s	(21H, 4.90)	3.1	CH_3	(24H, 1.78)
26	1Ha	3.12	m	(26He, 3.67)<br>(27Ha, 1.78)<br>(27He, 1.89)	49.8	CH_2	(26Ha, 3.12)
	1He	3.58	m	(26Ha, 3.10)<br>(27Ha, 1.78)<br>(27He, 1.89)	-	-	(26He, 3.58)
27	1Ha	1.71	m	(26Ha, 3.12)<br>(26He, 3.58)<br>(27He, 1.83)<br>(28Ha, 1.42)<br>(28He, 1.85)	23.1	CH_2	(27Ha, 1.71)
	1He	1.83	m	(26Ha, 3.12)<br>(26He, 3.58)<br>(27Ha, 1.71)<br>(28Ha, 1.42)<br>(28He, 1.85)	-	-	(27He, 1.83)
28	1Ha	1.42	m	(27Ha, 1.78)<br>(27He, 1.89)	29.9	CH_2	(28Ha, 1.42)

				(28He, 2.02)<br>(29H, 3.02)			
	1He	1.85	m	(27Ha, 1.71)<br>(27He, 1.83)<br>(28Ha, 1.42)<br>(29H, 3.68)	-	-	(28He, 1.85)
29	1H	3.68	m	(28Ha, 1.42)<br>(28He, 1.85)<br>(30Ha, 2.92)<br>(30He, 3.67)<br>(31H, 6.13)	45.5	CH	(29H, 3.68)
30	1Ha	2.92	m	(29H, 3.68)<br>(30He, 3.67)	54.6	CH_2	(30Ha, 2.92)
	1He	3.67	m	(29H, 3.68)<br>(30Ha, 2.92)	-	-	(30He, 3.67)
31	NH	6.13	d, 7.2	(29H, 3.68)	-	-	-
32	3H	3.51	s	-	29.4	CH_3	(32H, 3.51)
33	3H	2.90	s	-	21.6	CH_3	(33H, 2.90)
34	-	-	-	-	147.6	-	-
35	NH_2	5.48	s	-	-	-	-

[1] Refer the structural formula in previous page for numbering

[2]This column gives the ^{1}H-^{1}H multiplicity and coupling constants

s-singlet, d-doublet, t-triplet, m-multiplet.
